# Imaging the Ultrastructure of Isolated Peptidoglycan Sacculi from Rod-Shaped *Helicobacter pylori* J99 Cells by Atomic Force Microscopy

**DOI:** 10.3390/molecules30010155

**Published:** 2025-01-03

**Authors:** Daniel Amiteye, Jandirk Sendker, Fabian Herrmann

**Affiliations:** Institute of Pharmaceutical Biology and Phytochemistry, University of Münster, 48149 Münster, Germany; amiteye@uni-muenster.de (D.A.); jandirk.sendker@uni-muenster.de (J.S.)

**Keywords:** Atomic Force Microscopy, *Helicobacter pylori* J99, cell wall, peptidoglycan, ultrastructural imaging

## Abstract

Peptidoglycan is the basic structural polymer of the bacterial cell wall and maintains the shape and integrity of single cells. Despite years of research conducted on peptidoglycan’s chemical composition, the microscopic elucidation of its nanoscopic architecture still needs to be addressed more thoroughly to advance knowledge on bacterial physiology. Apart from the model organism *Escherichia coli*, ultrastructural imaging data on the murein architecture of Gram-negative bacteria is mostly missing today. This study therefore intended to further our understanding of bacterial physiology by the isolation of peptidoglycan sacculi from the Gram-negative bacterium *Helicobacter pylori* J99 and the subsequent nanoscopic imaging of the murein network by Atomic Force Microscopy (AFM). With the ability to purify peptidoglycan sacculi from *H. pylori* J99 for AFM by a modified peptidoglycan isolation protocol, nanoscopic imaging of the murein network by intermittent-contact AFM in air and under liquid yielded ultrastructural insights into the *H. pylori* J99 cell wall architecture. High-resolution data acquisition on isolated peptidoglycan from *H. pylori* J99 by AFM under liquid was performed and revealed a molecular network similar to available data from *E. coli*. Subsequent enzymatic digestion of the isolated *H. pylori* J99 sacculi and analysis of the resulting fragments by +ESI-LCMS confirmed the presence of *N*-acetylglucosamine as an additional marker for successful peptidoglycan isolation. By comparison of the nanoscopic sacculus dimensions of *H. pylori* J99 to *E. coli* NU14, this study also identified specific differences in the sacculus morphology of both Gram-negative pathogenic bacteria.

## 1. Introduction

*Helicobacter pylori* is a widespread human pathogen and one of the most common causes of infectious diseases worldwide [1,2]. It is estimated that almost 50% of the world’s population is infected by *H. pylori*, with 80–90% being adult patients [3]. The Gram-negative bacterium resides in the gastric mucosa of the human gastrointestinal tract, with an increased risk of gastric adenocarcinoma (1–3%), gastric lymphoma (<0.1%), and peptic ulcer disease (10–15%) [4]. Most individuals infected by *H. pylori* are asymptomatic [4,5]. The predominant morphological shape of this bacterium is spiral, which is essential for proper colonization of the stomach by enabling the corkscrew penetration of the dense gastric mucin layer [6]. However, the spiral form is not the only morphology occurring and there are also numerous heterogeneities including straight rod, filamentous, and coccoid forms [7]. Peptidoglycan is the fundamental structural polymer of the bacterial cell wall and plays an essential role in maintaining the shape and integrity of individual cells [8]. In addition, especially in times of rising bacterial resistance against clinically administered antibiotics, peptidoglycan and cell wall synthesis are of ever so high importance as targets for the development of novel antibiotics [9]. Several studies have shown that *H. pylori* cell morphology is closely associated with the peptidoglycan architecture [7,10]. Nevertheless, studies elucidating the ultrastructure of peptidoglycan sacculi from *H. pylori* are mostly missing today. The only study conducted on the morphology of isolated *H. pylori* peptidoglycan sacculi was not focused on the high-resolution imaging of the murein ultrastructure, although supplying transmission electron microscopy (TEM) data on isolated *H. pylori* sacculi. The high-quality study conducted by Salama and co-workers [7] was mainly aimed at the correlation of peptidoglycan architecture with the resulting morphological form of *H. pylori*.

The isolation of *Escherichia coli* peptidoglycan sacculi allowed a variety of insights into, e.g., its chemical composition in the past, but its architecture and dynamics of division and growth are still not fully understood [11,12]. Numerous models have been proposed for peptidoglycan architecture, including layered and framework models [13,14]. Nonetheless, microscopic observations on peptidoglycan, especially down to a lower nanoscale, are still mostly missing apart from studies on the model organism *E. coli* [12,13,14].

Besides the isolation of bacterial peptidoglycan, subsequent ultrastructural imaging of the murein network is a challenging process. Techniques like TEM have very often resulted in poor imaging contrast and rather low resolution not showing details of the molecular peptidoglycan network on isolated sacculi [15]. Currently, Atomic Force Microscopy (AFM) is the most promising technique to gain ultrastructural insights into peptidoglycan architecture. AFM is an imaging technique that allows direct insight into the topography of biological specimens at the lower nanoscale [16,17]. In particular, AFM-based ultrastructural data on live bacteria cells and isolated peptidoglycan sacculi have been reported in recent years [18,19,20,21,22,23]. Some AFM studies have even presented *E. coli* peptidoglycan image data with molecular resolution [12,23]. Although studies exist on the morphology of isolated murein sacculi from *H. pylori* using electron microscopy [15], ultrastructural imaging data of the architecture of the murein network from the Gram-negative bacterium *H. pylori* is mostly missing today.

Hence, in this study, peptidoglycan sacculi from *H. pylori* J99 (ATCC 700824) suspension cultures were isolated by a modified purification protocol and subsequently imaged by intermittent-contact mode AFM in air and liquid, enabling the nanoscopic depiction of *H. pylori* J99 sacculus dimensions and peptidoglycan architecture.

Because of the high variability and the occurring transition between different *H. pylori* morphologies in high-passage in vitro experiments, this study focused on the ultrastructural imaging of isolated peptidoglycan sacculi from rod-shaped *H. pylori* J99 cells only. This was mainly intended to ensure the reproducibility of nanoscopic peptidoglycan imaging of *H. pylori* J99 sacculi.

Measurements of morphological characteristics from rod-shaped *H. pylori* J99 murein sacculi were carried out and compared to those of the clinically relevant pathogen *E. coli* NU14. The analysis of the peptidoglycan morphology of both species revealed specific differences in murein ultrastructure, indicating physiological differences between *H. pylori* J99 and *E. coli* NU14 cell wall architecture.

The experimental techniques described in this article altogether allowed easy and reproducible insight by AFM into the overall dimensions as well as the ultrastructure of *H. pylori* J99 and *E. coli* NU14 peptidoglycan sacculi. With the presented protocols of this publication, researchers in the field of peptidoglycan analysis have a facile tool at hand, not only allowing the investigation of peptidoglycan morphology but subsequently also enabling the evaluation or potentially even the identification of compounds affecting peptidoglycan synthesis in *H. pylori* and other Gram-negative bacteria. Even today, experimental proof of an activity of an antibacterial compound on peptidoglycan synthesis is highly difficult to assess (e.g., by commonly used “omic” techniques, or enzyme inhibition experiments). Our approach presented here will at least add another option to investigate activity against peptidoglycan synthesis.

## 2. Results

### 2.1. H. pylori J99 Culture and Morphology

**Figure 1:**
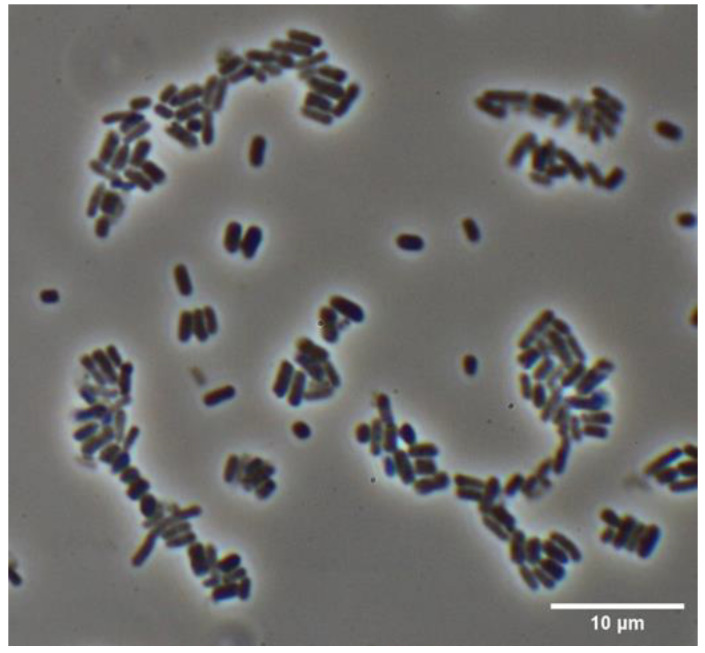
Representative phase-contrast light microscopic image of a *H. pylori* J99 suspension culture at an OD_600_ of 0.2. The predominant cell shape under typical high-passage in vitro cultivation conditions was a rod-like morphology.

*H. pylori* J99 (ATCC 700824) cells were cultured in Brucella broth with 10% fetal calf serum (FCS) supplemented with CampyGen at 37 °C overnight under agitation at 220 rpm. Subsequently, the morphology of *H. pylori* J99 cells was determined in three biological replicates after the cells were cultivated to the mid-exponential phase (OD_600_ 0.2 to 0.3) and assessed using high-magnification phase-contrast microscopy (Figure 1). The investigated cells showed overall typical rod-shaped native cell morphology as described previously in high-passage in vitro suspension cultures of *H. pylori* [24] (Figure 1). However, as the name Helicobacter implies, its morphology is also specifically known to be spiral, besides commonly occurring rod- or spherical-shaped cell forms [25,26]. Little is known about the environmental conditions that stimulate the transition to rod-shaped morphology, whereas it is observed that in fresh clinical isolates of *H. pylori* about 10–15% of all cells occur in rod-shaped forms [25]. During high-passage in vitro cultivation, the number of rod-shaped individuals increases, with an inverse correlation observed in the number of spiral forms. A drastic change in the culture environment from a nutrient-rich medium to pure water or saline solution contributes to the transformation of *H. pylori* cells to spherical forms [27,28]. In order to allow reproducible insights into the ultrastructural morphology of isolated peptidoglycan sacculi from *H. pylori* J99 suspension cultures, and due to the easy comparability to other Gram-negative bacteria, the present study focused on the nanoscopic analysis of rod-shaped murein isolates from *H. pylori* J99.

### 2.2. Isolation and Purification of Peptidoglycan Sacculi from H. pylori J99 Suspension Cultures

Successful isolation of murein sacculi from bacterial suspension cultures, e.g., for subsequent chromatographic analysis, has been carried out for decades [29]. This was mainly performed by incubation in boiling solutions of sodium dodecyl sulfate (SDS) in the past, due to the intrinsic sacculus stability and its insensitivity to solvation by detergent treatment [29,30,31]. However, these techniques were not yet applied to *H. pylori* and subsequent ultrastructural imaging by AFM. In order to mediate AFM-based insights into peptidoglycan architecture from *H. pylori* J99, the previously described isolation techniques [29] were adapted to meet AFM imaging requirements. To do so, especially the introduction of a final purification step using 1% (*v*/*v*) SDS solution was needed to remove the previously added enzymes (*amylase* and *pronase E*) as well as remaining sugars and proteins. Additionally, the concentration of the initial SDS treatment was adjusted to 8% instead of 4% as reported by the literature [29], in order to separate and remove all the soluble cell components from insoluble macromolecular peptidoglycan, since 4% SDS could not eliminate remnants entirely in the case of *H. pylori* J99 sacculus isolation (Figure S1 of Supplementary Materials). The mentioned modifications reproducibly yielded almost remnant-free and easy-to-immobilize *H. pylori* J99 murein sacculi ready for high-resolution AFM imaging.

### 2.3. Imaging of Isolated H. pylori J99 Peptidoglycan Sacculi by Intermittent-Contact Mode AFM in Air

**Figure 2:**
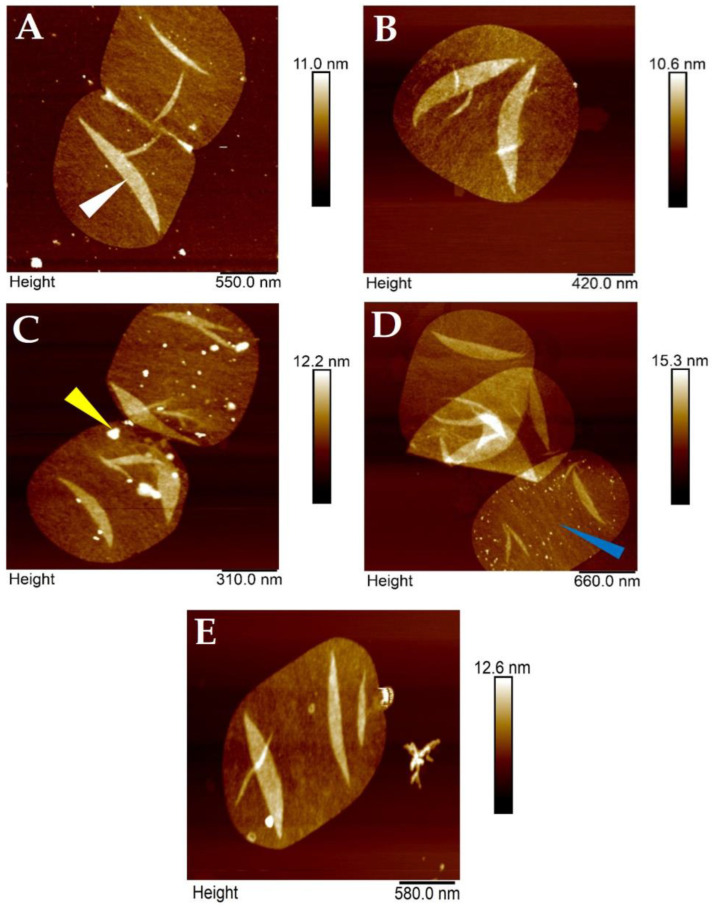
Representative AFM images of isolated, purified, and immobilized *H. Pylori* J99 (**A**–**D**) and *E. coli* NU14 (**E**) peptidoglycan sacculi recorded by intermittent-contact mode AFM under ambient conditions in air. Intermittent-contact mode AFM imaging in air yielded valuable ultrastructural insights into general *H. Pylori* J99 peptidoglycan sacculus morphology. The expected nanoscopic murein structure with typical septal morphology (exemplary septum marked with white arrow in (**A**)) and delicate bands with perpendicular orientation to the growth axis of single bacteria cells (blue arrow in (**D**)) were clearly recognized. Note the overall rather low number of intracellular remnants as a result of the modified isolation protocol employed for *H. Pylori* J99 sacculus purification (representative intracellular remnant marked by yellow arrow in (**C**)). A representative peptidoglycan sacculus from *E. coli* NU14 of this study is shown in (**E**).

**Figure 3:**
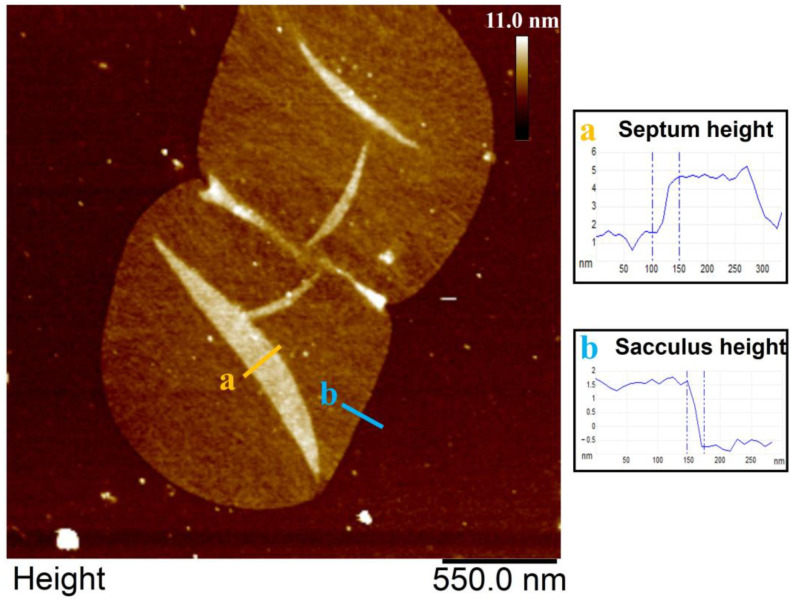
Representative morphological measurements on an isolated, purified, and immobilized *H. Pylori* J99 sacculus employing data collected by intermittent-contact mode AFM in air. Measurements are represented by typical section planes for each analyzed parameter (a and b). Dashed blue lines in a and b indicate reference points for the different measurements. Prior to any sectioning measurement, AFM images were flattened employing the 0th-order algorithm implemented in the software Nanoscope Analysis 3.0 (Bruker, Karlsruhe, Germany).

**Figure 4:**
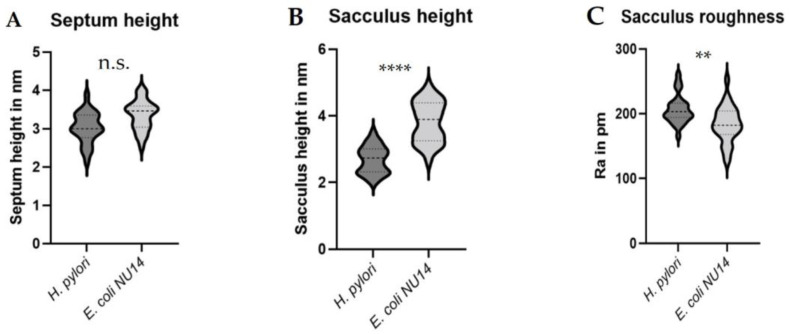
Violin plots representing measurements of morphological characteristics from isolated murein sacculi of *H. pylori* J99 compared to corresponding measurements on *E. coli* NU14 sacculi prepared by the same isolation protocol (**A**–**C**). Altogether, nine isolated sacculi from both species were analyzed for each parameter (three sacculi for each of the three replicates per species). Each parameter was measured at least thrice for every sacculus. Statistical variance was analyzed employing the Student t-test algorithm implemented in the software GraphPad Prism 9 (n.s. = no significant difference, ** = *p* < 0.01, **** = *p* < 0.0001; GraphPad Prism 9, GraphPad Software, Boston, MA, USA). No significant differences were detected between the overall septum heights of *H. pylori* J99 and *E. coli* NU14 (**A**). Measurements of sacculus height (**B**) as well as sacculus roughness (**C**) data showed significant differences between the two Gram-negative species, indicating differing physiology of their peptidoglycan architecture and/or composition.

Intermittent-contact mode AFM in air of isolated and immobilized *H. pylori* J99 sacculi subsequently provided a nanoscale view of the sacculus peptidoglycan architecture with lateral resolution of about 5 nm and vertical resolution below 1 nm. AFM imaging of *H. pylori* J99 murein sacculi under ambient conditions in air was able to resolve bands of peptidoglycan layers perpendicular to the growth axis, without individual peptidoglycan strands being visualized (Figure 2A–D). At this level of resolution, the murein network of purified *H. pylori* J99 sacculi appeared to be evenly distributed overall, also showing less and more dense areas of peptidoglycan (Figure 2A–D). At the poles of single sacculi, septal remains were frequently recognizable. The isolated and immobilized sacculi proved to be easily scannable by intermittent-contact mode AFM in air and allowed facile recording of a larger number of images on a high number of different sacculi from *H. pylori* J99. Based on this dataset, a subsequent detailed analysis of morphological characteristics from H. pylori J99 sacculi compared to isolates from *E. coli* NU14 became available (Figure 3 and Figure 4).

### 2.4. Comparing Morphological Characteristics of Isolated Peptidoglycan Sacculi from H. pylori J99 and E. coli NU14

One main advantage of AFM-based imaging is the highly accurate z-axis data obtained by this microscopic technique. In the case of the isolated peptidoglycan sacculi from *H. pylori* J99 and *E. coli* NU14 from this study, the acquired AFM data allowed detailed insights into the overall dimensions of *H. pylori* J99 peptidoglycan sacculi as well as a comparison to a variety of morphological measurements on isolated sacculi from *E. coli* NU14 (Figure 3 and Figure 4). In detail, the mentioned AFM data enabled the comparison of the overall sacculus height, the septum height, and the roughness of peptidoglycan from *E. coli* NU14 to corresponding *H. pylori* J99 morphology. Three morphological parameters showed significant differences in the carried-out z-axis measurements between both species (septum height, sacculus height, and roughness, Figure 4A–C. Especially the differing sacculus and septum heights of *H. pylori* J99 and *E. coli* NU14 could be explained due to alterations in the peptidoglycan organization, indicating different murein physiology in the two Gram-negative species. This hypothesis is also supported by the measured significant differences in sacculus roughness (Figure 4C).

In conclusion, AFM-based imaging of isolated *H. pylori* J99 murein sacculi under ambient conditions in air yielded specific insights into the general peptidoglycan architecture of the J99 subtype and enabled detailed comparison to the sacculus morphology from *E. coli* NU14 (Figure 3). This approach revealed differences in overall sacculus morphology between the two assessed bacterial species and potentially indicates differences in overall peptidoglycan architecture and/or composition. In order to ensure that measured roughness parameters of the isolated sacculi were not somehow correlated to the roughness values of whole bacteria, Ra assessments were also carried out on complete *H. pylori* J99 and *E. coli* NU14 cells (Figures S2 and S3 of Supplementary Materials).

### 2.5. High-Resolution AFM Imaging of Isolated H. pylori J99 Peptidoglycan Sacculi by Intermittent-Contact Mode in Liquid

**Figure 5:**
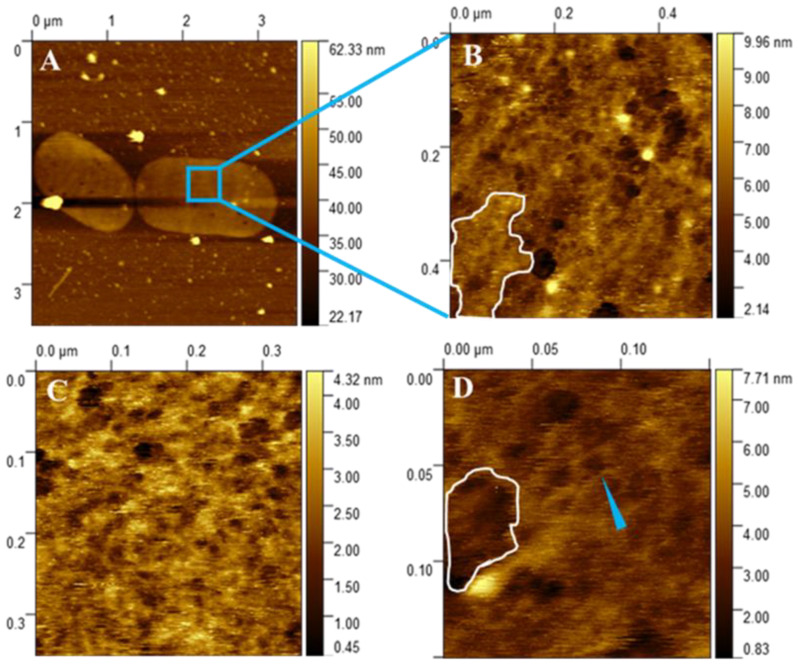
Representative AFM height channel images of isolated *H. pylori* J99 peptidoglycan sacculi imaged by intermittent-contact mode AFM under liquid conditions. The modified isolation protocol of this study reproducibly enabled lower nm resolution on isolated sacculi from *H. pylori* J99, although true molecular resolution on single peptidoglycan strands was not reached. Nanoscopic insights into the peptidoglycan architecture of *H. pylori* J99 were established by AFM imaging in TRIS/HCl buffer. Representative image of two complete sacculi is shown in (**A**), details in images (**B**–**D**). Note the evenly distributed peptidoglycan network consisting of crosslinked macromolecules ((**B**–**D**), single strand-like macromolecule indicated with blue arrow in (**D**)). Additionally, typical dense and less dense regions of peptidoglycan architecture were recognized in the AFM data, attributable to the highly dynamic ultrastructure of peptidoglycan (more dense area highlighted in (**B**), less dense area highlighted in (**D**)).

To gain more detailed access to the architecture of isolated *H. pylori* J99 peptidoglycan sacculi, intermittent-contact AFM imaging was also performed on isolated and immobilized sacculi under liquid conditions (imaging buffer 10 mM TRIS/HCl at pH 8). The observation of the general morphology of the sacculi in liquid was altogether consistent with the previous data obtained from the measurements in air (Figure 2). However, the significantly higher resolution potential of peptidoglycan measurements in liquid allowed a more detailed view of the ultrastructural morphology of single sacculi. High-resolution AFM revealed randomly oriented interconnected strands (Figure 5B–D). Corresponding to the dynamic nature of bacterial murein sacculi [12], more and less dense regions of peptidoglycan were also often recognized in the ultrastructural AFM data (highlighted areas in Figure 5B,D).

The data acquired in this study by AFM on immobilized sacculi in liquid altogether allowed detailed nanoscopic insights into *H. pylori* J99 murein architecture, showing filamentous peptidoglycan strands on the lower nanometer scale (Figure 5D). These data were highly comparable to the AFM imaging data on *E. coli* peptidoglycan sacculi from the available literature [12,23]. To the best knowledge of the authors, the image data of this study represent the highest microscopic resolution on isolated peptidoglycan sacculi from *H. pylori* reported so far.

The ability to investigate the *H. pylori* J99 peptidoglycan architecture on this ultrastructural level might not only enable new insights into Gram-negative cell wall physiology of this pathologically highly relevant species in the future. Additionally, these techniques may also advance the evaluation of compounds directly targeting peptidoglycan synthesis and/or assembly in *H. pylori*, potentially also mediating antibiotic development in the future.

### 2.6. Enzymatic Digestion of H. pylori J99 Sacculi and Subsequent +ESI-LCMS Analysis of the Resulting Muropeptide Fragments

**Figure 6:**
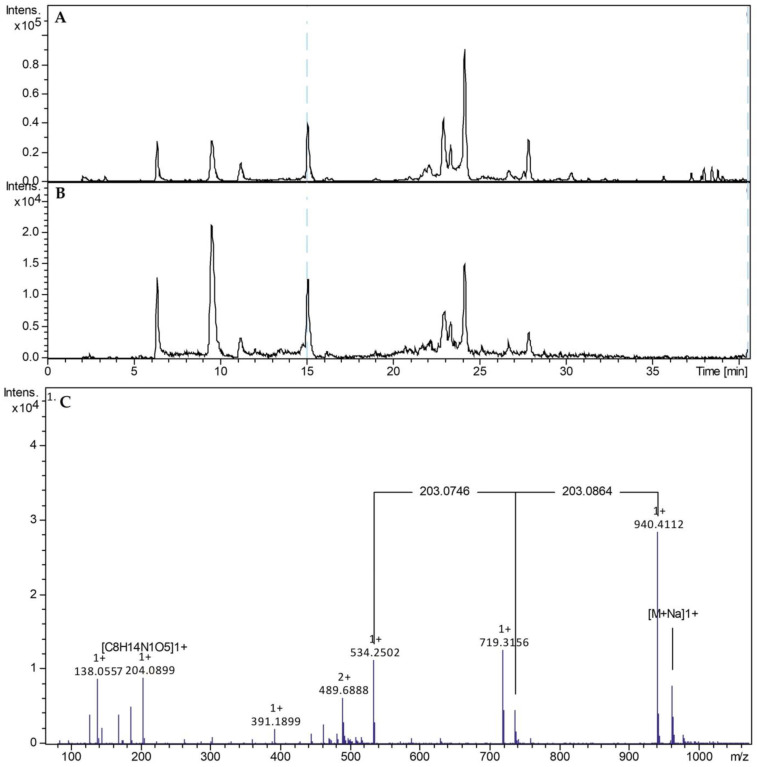
+ESI-LCMS of digested muropeptide of *Helicobacter pylori* J99. (**A**): Overview base peak chromatogram, full mass range (*m/z* 50–1500). (**B**): Extracted ion chromatogram indicative of the presence of the *N*-acetylglucosamine fragment at *m/z* 204.0866 [C_8_H_13_NO_5_ + H]^+^. (**C**): +ESI-MS spectrum of the peak at 15 min. The theoretical ion mass of the protonated *N*-acteylglucosamine-fragment [C_8_H_14_NO_5_]^+^ deviates from the measured ion mass of 204.0899 u by 3.2 mu, mΣ is 9.4.

In order to prove the successful isolation of H. pylori J99 peptidoglycan sacculi on a chemical basis, the isolated sacculi were enzymatically digested (mutanolysin from *Streptomyces globisporus*) and subsequently analyzed by LC-MS. +ESI-LCMS of the digested sacculi showed in the overview chromatogram a pattern of peaks (Figure 6A), each of which showed indications of the presence of N-acetylglucosamine moieties by neutral losses of 203 u and the signal of the protonated N-actylglucosamine fragment at *m*/*z* 204 (Figure 6B). An exemplary spectrum is shown in Figure 6C. In this spectrum, two neutral losses of N-actylglucosamine connect the protonated molecule with the fragment signal at *m*/*z* 534, which gives no indication of further loss of N-actylglucosamine and is thus assumed to be a peptidic cross-linkage. No information on the composition of this molecule part could be gained from this preliminary analysis.

## 3. Discussion

This study aimed at the further elucidation of the nanoscopic *H. pylori* J99 peptidoglycan architecture employing a modified murein sacculus isolation technique and subsequent nanoscopic AFM imaging in air and under liquid conditions, as well as the chemical analysis of enzymatically digested muropeptide fragments by +ESI-LCMS.

The modification of commonly available peptidoglycan isolation protocols reported in this article reproducibly allowed the purification of nearly remnant-free *H. pylori* J99 sacculi suitable for high-resolution AFM imaging. Especially the application of an initial (8%) and final (1%) SDS solubilization step before and after *pronase* and *amylase* digestion was key to the sufficient isolation of *H. pylori* J99 peptidoglycan without the presence of relevant intracellular protein or polysaccharide remnants impairing ultrastructural imaging. This isolation technique not only reproducibly yielded purified peptidoglycan sacculi but also proved to be easily carried out within a single lab day. Up to today, ultrastructural data of isolated *H. pylori* peptidoglycan has only been reported following TEM experiments without achieving resolutions on the lower nanometer scale [7].

After successful isolation, peptidoglycans from *H. pylori* J99 were enzymatically digested and analyzed by +ESI-LCMS. The analysis of the chromatographically separated muropeptides clearly revealed the presence of *N*-acetylglucosamine fragments at *m*/*z* values around 204, indicating the repeated elimination of *N*-acetylglucosamine from larger muropeptide chains. With the ability to additionally analyze the chemical composition of muropeptides isolated by our modified isolation technique, closer insights into *H. pylori* sacculus composition and architecture might be enabled in the future.

The intermittent-contact mode AFM initially performed in air in this study already allowed nanoscopic insights into immobilized *H. pylori* J99 peptidoglycan sacculi and enabled the precise assessment of morphological characteristics as well as the comparison of these data with similar measurements from isolated *E. coli* NU14 sacculi (sacculus height and roughness, septum height; Figure 4). Comparison of the recorded datasets on sacculus height and roughness as well as on septum height indicated differences in *H. pylori* J99 and *E. coli* NU14 peptidoglycan architecture, previously described by other experimental techniques [32]. Comparable thickness differences in AFM peptidoglycan data have also been reported for *E. coli* K12 and *Pseudomonas aeruginosa* by Yao et al. [33] and were attributed to missing lipoproteins of the outer membrane of *P. aeruginosa*, potentially resulting in overall reduced sacculus thickness [33]. In the case of *H. pylori* J99, previous studies have also indicated the absence of murein-bound lipoprotein and additionally shown increased levels of muropeptides with a pentapeptide chain compared to *E. coli* peptidoglycan architecture [32]. The mentioned alterations in *H. pylori* peptidoglycan morphology compared to the model organism *E. coli* could be considered as one potential explanation for the different height measurements of isolated murein sacculi reported in this study. Apart from insights into *H. pylori* J99 cell wall physiology and the general comparison to peptidoglycan data from other bacteria, the easy assessment of the mentioned ultrastructural details by AFM in air might also enable the analysis of effects on the cell wall assembly and synthesis in *H. pylori* J99 to some extent, as have been previously described for the model organism *E. coli* after antibiotic treatment [23].

With the ability to image isolated *H. pylori* J99 sacculi by AFM under liquid conditions, a substantially higher resolution was achieved on the murein network of this clinically highly relevant microorganism. At this magnification level, the imaged peptidoglycan sacculi clearly revealed bandlike interconnected structures previously reported for isolated peptidoglycan sacculi from *E. coli* [12,23]. Although not comprehensively reaching molecular resolution down to the level of single peptidoglycan strands by intermittent-contact AFM in the present study, the achieved magnification allowed previously unreported insights into the nanoscopic architecture of *H. pylori* J99 peptidoglycan. The data presented in this study clearly revealed network-like interconnected macromolecules as well as typical dense and less dense regions of highly dynamic peptidoglycan (Figure 5B–D), as has been reported for the model organism *E. coli* [12,23]. Because of the currently largely missing nanoscopic data on peptidoglycan architecture from bacteria other than *E. coli*, the interpretation of AFM sacculus images from other bacteria in liquid is still a challenging task. In comparison to the available molecular AFM data on *E. coli* peptidoglycan reported previously [12,23], no apparent differences between the overall network organization and our data from *H. pylori* J99 were recognizable.

## 4. Materials and Methods

### 4.1. Origin of Chemicals

If not stated otherwise, all employed chemicals were purchased from Merck KGaA (Darmstadt, Germany).

### 4.2. Bacterial Strains and Growth Conditions

The employed *H. pylori* J99 (ATCC^®^ 700824) strain glycerol stock was stored at −80 °C. Initially, 120 μL of the thawed stock solution was plated on trypticase soy blood agar using an inoculation spreader. The inoculated plate was incubated for 48 h at 37 °C under microaerophilic conditions (5% O_2_, 10% CO_2_, 85% N_2_), which were created by placing a 2.5 L CampyGenTM sachet in a sealed jar. After incubation, a single colony was harvested from the agar with a flame-sterilized metal loop and suspended into 10 mL of Brucella medium supplemented with 10% fetal calf serum (FCS). The suspension culture was incubated overnight at 37 °C and 220 rpm under microaerophilic conditions in anaerobically sealed jars with CampyGenTM sachets. The described overnight culture was subsequently diluted down to an OD_600_ of 0.02 with fresh Brucella medium supplemented with 10% FCS. Finally, 500 µL of this suspension culture was used for inoculation of 50 mL Brucella medium supplied with 10% FCS.

A total of 120 µL of a glycerol cryo-stock suspension of *E. coli* NU14 (NCBI txid569579 strain) stored at −80 °C was spread and cultivated for 24 h at 37 °C on lysogeny broth (LB) agar plates. A single colony was subsequently picked from the agar and used to prepare an overnight suspension culture by the inoculation of 10 mL of preheated LB medium overnight at 37 °C and 220 rpm. The described overnight culture was subsequently diluted down with fresh LB medium to an OD_600_ of 0.02 and 500 µL was used to inoculate 50 mL of preheated LB medium. The suspension cultures were afterwards cultivated under the above-mentioned growth conditions for subsequent peptidoglycan isolation.

### 4.3. Phase-Contrast Light Microscopy

For phase-contrast microscopy, a Leica Orthoplan research microscope (Leica Microsystems GmbH, Wetzlar, Germany) equipped with high-resolution phase-contrast optics (Leica PlAPO 63/1.4 Ph4) was used. Images were captured with a full-frame DSLR (Canon EOS 5D II, Canon, Tokyo, Japan).

### 4.4. Isolation of Peptidoglycan Sacculi from H. pylori J99 and E. coli NU14

Peptidoglycan sacculi were isolated and purified from *H. pylori* J99 and *E. coli* NU14 suspension cultures by a modified protocol previously described for *E. coli* [29]. Initially, 50 mL of suspension culture was inoculated and incubated until reaching exponential phase at an optical density (OD_600_) of 0.2 to 0.3. The suspension was afterwards centrifuged (10.000 rcf for 10 min) and the supernatant was discarded. The resulting pellet was resuspended in 3 mL of PBS, and the suspension was added dropwise to 5 mL of boiling 8% (*v*/*v*) SDS solution and agitated at 95 °C and 350 rpm for altogether 6 h. The resulting preparation was repeatedly washed with Aqua Millipore by centrifugation until no bubbles from SDS remnants were recognized anymore during washing. After a final washing step, the resulting precipitate was resuspended in 500 µL of 10 mM TRIS-HCl buffer (pH 7.2, 37 °C) containing α-amylase (100 μg/mL) and was incubated for 60 min. After another centrifugation, the supernatant was discarded again, the resulting pellet was resuspended in 500 µL of 10 mM TRIS-HCl buffer (pH 7.2, 60 °C) containing pronase-E (100 µg/mL) and incubated for another 90 min. In the following purification step, the TRIS-HCl buffer was removed by centrifugation, and the resulting pellet was resuspended in 3 mL of 1% (*v*/*v*) SDS solution to remove the added enzymes and the solubilized polysaccharide and protein remnants. The SDS was afterwards removed again by washing with Aqua Millipore until no bubbles from SDS remnants were recognized and the isolated sacculi were finally resuspended in Aqua Millipore and stored at 4 °C for subsequent preparation for AFM imaging.

### 4.5. Preparation of Isolated Peptidoglycan Sacculi for AFM Intermittent-Contact Mode Imaging Under Ambient Conditions in Air

A circular 15 mm V1 mica disc (Nanoandmore GmbH, Wetzlar, Germany) was initially glued to a standard glass slide and cleaved at least three times using adhesive tape. Subsequently, 100 µL of peptidoglycan suspension was pipetted onto the mica disc and allowed to dry completely. The dried spot was afterwards covered with 100 μL of distilled water and allowed to settle for 15 min. The added water layer was then removed by hand bellow blowing until complete dryness of the preparation. Peptidoglycan sacculi prepared on mica discs according to the described protocol proved to be easily scannable by intermittent-contact mode AFM in air. Subsequent microscopic evaluations of the dried preparations were carried out with a Bruker Dimension 3100 AFM equipped with a Nanoscope IIIa controller and soft intermittent-contact mode silicon cantilevers (HQ:NSC14 Al/BS, µmasch, Sofia, Bulgaria). The samples were imaged at frequencies 40% below the cantilever’s resonance, employing RMS amplitudes around 0.8 V, amplitude setpoints around 0.6 V, and a scanning rate of 0.5 Hz.

### 4.6. Preparation of Isolated Peptidoglycan Sacculi for Intermittent-Contact Mode AFM Imaging Under Ambient Conditions in Liquid

To obtain nanoscopic images of isolated peptidoglycan sacculi in liquid, a circular 15 mm V1 mica disc (Nanoandmore, Wetzlar, Germany) was affixed to a standard 15 mm steel support disc with UV-curable epoxy resin (3D Rapid Printer Model Resin Clear, Monocure 3D, Sydney, Australia). The immobilized mica disc was then cleaved at least three times with adhesive tape and afterwards coated with poly-D-lysine (100 µL of a 0.1 mg/mL poly-D-lysine solution in Aqua Millipore, incubated for 30 min) to promote adhesion of the isolated peptidoglycan sacculi to the mica substrate. Finally, the mica surface was washed thoroughly with 100 µL of Aqua Millipore at least three times and dried under continuous air flow. A 100 µL sample volume of the suspension containing peptidoglycan isolates described above was washed once with 10 mM TRIS pH 8 (imaging buffer) and resuspended in 400 µL imaging buffer. Next, 100 µL of sacculus suspension was applied to the coated mica discs and allowed to rest for 60 min without drying. After the incubation, the sample was washed repeatedly with 100 µL of imaging buffer. During the entire sample preparation for liquid imaging, the samples were never allowed to dry. Finally, the mica discs were covered in imaging buffer and stored in a petri dish until measured by intermittent-contact mode AFM in liquid. Imaging was performed employing an Asylum Research Cypher-S AFM connected to an SPM ARC2 controller (Asylum Research, Oxford Instruments, Santa Barbara, CA, USA) in 10 mM TRIS/HCl buffer at pH 8. Standard PNP-TR probes were used for all measurements in liquid (Tip A, nominal resonance frequency in air 67 kHz, nominal spring constant of 0.32 N/m, and a nominal tip radius of curvature below 10 nm, NanoWorld, Neuenburg, Switzerland). The imaging resonance frequency was initially specified by the thermal noise tuning algorithm implemented in the Cypher-S AFM operating software (Igor Pro 6.3.8.1, Asylum Research, Oxford Instruments, Santa Barbara, CA, USA) and was subsequently adjusted by manual tuning close to the sample’s surface. The RMS amplitude was typically set to values between 0.5V and 0.7 V and imaging was carried out at amplitude setpoints below around 20% of the RMS amplitude employing scanning rates of 0.5 Hz.

### 4.7. Analysis of AFM Imaging Data Obtained Under Ambient Conditions in Air

The AFM data from measurements in air were completely processed with the software Nanoscope Analysis 3.0 (Bruker, Karlsruhe, Germany). Images were initially flattened (typically, 0th-order algorithms) before performing any measurements on the AFM data.

### 4.8. Measurements and Statistical Analysis of Nanoscopic H. pylori J99 Sacculus Morphology and Comparison to Data from E. coli NU14 (Sacculus Height and Roughness as Well as Septum Height)

Measurements on peptidoglycan sacculus morphology were carried out on AFM height data obtained under ambient conditions in air employing the section analysis tool of the software NanoScope Analysis 3.0 (Bruker, Karlsruhe, Germany). For the determination of sacculus and septum height, data sections on the boarders of remnant-free septae and sacculi as well as subsequent height measurements were carried out thrice for nine different sacculi and septae from *H. pylori* J99 and *E. coli* NU14 (see Figure 4 for details). Additionally, the average roughness (Ra) of isolated peptidoglycan was measured thrice for each of altogether nine different sacculi from *H. pylori* J99 and *E. coli* NU14 employing measurement areas of 150 by 150 nm. Statistical evaluation and comparative violin plots of the mentioned measurements were subsequently created using the software GraphPad Prism 9 (GraphPad Software, Boston, MA, USA).

### 4.9. Analysis of AFM Imaging Data Obtained Under Ambient Conditions in TRIS/HCl Buffer

AFM images obtained by measurements in liquid were analyzed with the open-source software Gwyddion 2.58v [34]. Typically, AFM images were initially flattened (0th- and 1st-order algorithms) and threshold values were adjusted according to the most favorable representation of recorded height data.

### 4.10. Enzymatic Digestion of H. pylori J99 Peptidoglycan Sacculi

Enzymatic digestion of the isolated H. pylori J99 sacculi was performed by the application of mutanolysin from *Streptomyces globisporus* (Merck, Darmstadt, Germany) as follows: Initially, 100 µL of peptidoglycan suspension was transferred into an Eppendorf reagent tube (1.5 mL) and 5 µL of mutanolysin solution (5000 U/mL) was added and incubated at 37 °C and 350 rpm for 20 h. After incubation, the added enzyme mixture was inactivated by heating the reagent tube at 99 °C for another 5 min. The solution was finally centrifuged at 20,000 rcf for 10 min and the supernatant subsequently used for +ESI-LCMS analysis.

### 4.11. +ESI-LCMS Analysis of Enzymatically Digested Muropeptides from H. pylori J99

Chromatographic separations were performed on a Dionex Ultimate 3000 RS Liquid Chromatography System on a Waters (Milford, MA, USA) Aquity UPLC^®^ HSS T3, 1.8 µm, 2.1 × 100 mm column with a binary gradient (A: water with 0.1% formic acid; B: acetonitrile with 0.1% formic acid) at 0.2 mL/min: 0 to 30 min: linear from 0% B to 30% B; 30 to 38 min: curve 7 from 30% B to 80% B; 38 to 38.5 min: linear from 80% B to 100% B; 38.5 to 45 min: isocratic at 100% B; 45 to 45.1 min: linear from 100% B to 0% B; 45.1 to 55 min: isocratic at 0% B. The injection volume was 50 µL. Eluted compounds were detected using a Bruker Daltonics micrOTOF-QII time-of-flight mass spectrometer equipped with an Apollo electrospray ionization source in positive mode at 2 Hz over a mass range of *m/z* 80–1500 using the following instrument settings: nebulizer gas nitrogen, 3 bar; dry gas nitrogen, 9 L/min, 220 °C; capillary voltage, 4500 V; end plate offset, −500 V; transfer time, 70 µs; prepulse storage, 6 µs; collision gas, nitrogen; collision energy, 7 eV (MS1); collision RF, 130 Vpp. Internal dataset calibration (HPC mode) was performed for each analysis using the mass spectrum of a 10 mM solution of sodium formiate in 50% isopropanol that was infused during LC re-equilibration using a divert valve equipped with a 20 µL sample loop. Data were analyzed using Bruker DataAnalysis 4.1 SP1.

## Data Availability

Data presented in this manuscript are available from the authors by request.

## Supplementary Materials

The following supporting information can be downloaded at: https://www.mdpi.com/article/10.3390/molecules30010155/s1, Figure S1: Exemplary image of *Helicobacter pylori* J99 sacculi isolated by the traditional protocol (one initial 4% SDS step, no final SDS step).; Figure S2: Exemplary images of a *Helicobacter pylori* J99 (left) and an *Escherichia coli* NU14 cell (right).; Figure S3: Representation of roughness measurements of dried in *H. pylori* J99 and *E. coli* NU14 cells (nine cells for each species, three roughness measurements for each cell).
