# Supplementary material for: Imaging the Ultrastructure of Isolated Peptidoglycan Sacculi from Rod-Shaped Helicobacter pylori J99 Cells by Atomic Force Microscopy
Source: Molecules. 2025 Jan 3;30(1):155. doi: 10.3390/molecules30010155 (PMC11720842; doi:10.3390/molecules30010155)
Supplement: Supplementary file 1 [file molecules-30-00155-s001.zip › molecules-3195226-supplementary.pdf]

Supplementary material – Imaging the ultrastructure of isolated peptidoglycan sacculi from rod-shaped *Helicobacter pylori* J99 cells by Atomic Force Microscopy

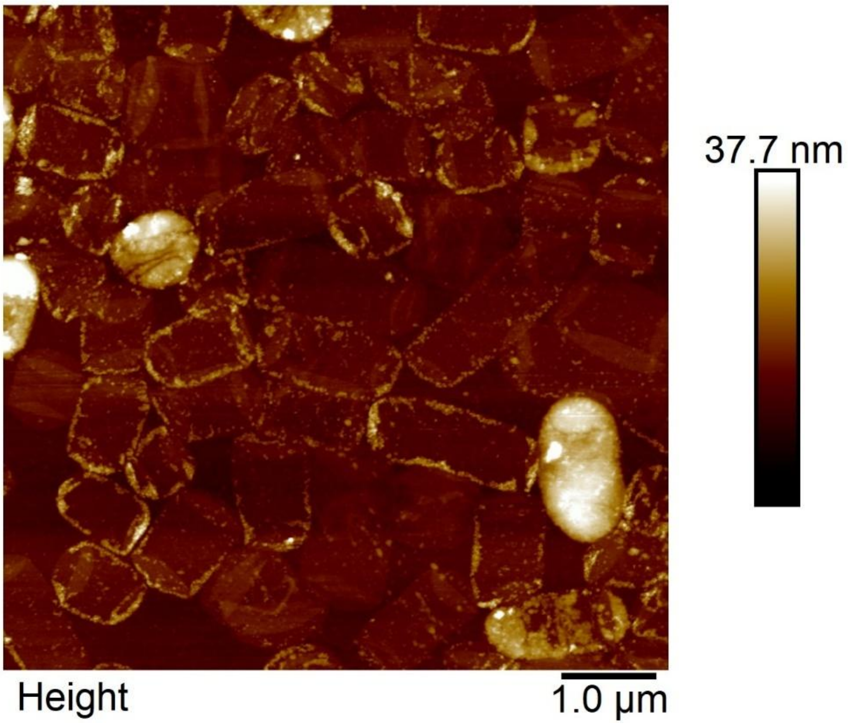

Figure S1: Exemplary image of *Helicobacter pylori* J99 sacculi isolated by the traditional protocol (one initial 4% SDS step, no final SDS step). Please note abundant remnants remaining in the sacculi, leading to generalized problems during AFM data acquisition.

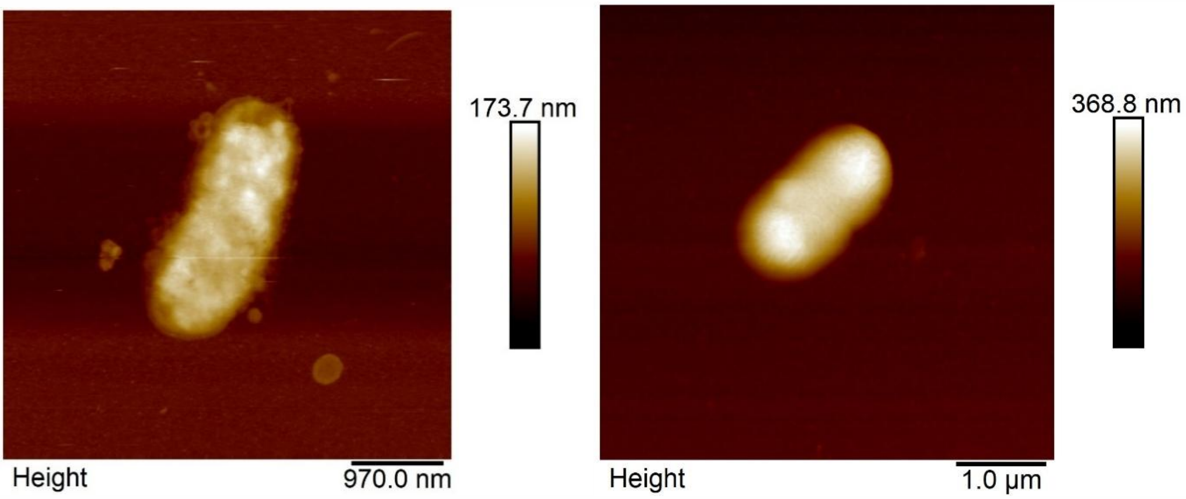

Figure S2: Exemplary images of a *Helicobacter pylori* J99 (left) and an *Escherichia coli* NU14 cell (right). Please note the representative height of the cells below 200 nm (*H. pylori* J99) and below 400 nm (*E. coli* NU14). An isolated peptidoglycan sacculus typically shows a height below 5 nm.

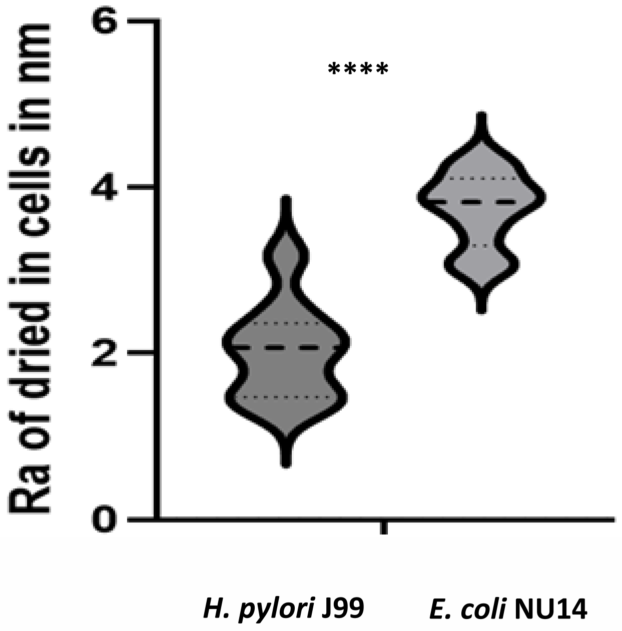

Figure S3: Representation of roughness measurements of dried in *H. pylori* J99 and *E. coli* NU14 cells (nine cells for each species, three roughness measurements for each cell). The roughness of dried in cells significantly differs between H. pylori J99 and E. coli NU14 as expected. Nevertheless, the overall roughness of dried in cells is far higher compared to the Ra of isolated sacculi (nm range in bacteria, pm range in sacculi). A potential correlation of the roughness of dried in cells with the corresponding isolated peptidoglycan sacculi can be rationally excluded at this point. **** = $p < 0.0001$.
